# Stat3: linking inflammation to epithelial cancer - more than a "gut" feeling?

**DOI:** 10.1186/1747-1028-5-14

**Published:** 2010-05-17

**Authors:** Andrew Jarnicki, Tracy Putoczki, Matthias Ernst

**Affiliations:** 1 Ludwig Institute for Cancer Research, PO Box 2008 Royal Melbourne Hospital, VIC 3050, Australia

## Abstract

Inflammation is an important environmental factor that promotes tumourigenesis and the progression of established cancerous lesions, and recent studies have started to dissect the mechanisms linking the two pathologies. These inflammatory and infectious conditions trigger immune and stromal cell release of soluble mediators which facilitate survival and proliferation of tumour cells in a paracrine manner. In addition, (epi-)genetic mutations affecting oncogenes, tumour-suppressor genes, chromosomal rearrangements and amplifications trigger the release of inflammatory mediators within the tumour microenvironment to promote neoplastic growth in an autocrine manner. These two pathways converge in tumour cells and result in activation of the latent signal transducer and activator of transcription 3 (Stat3) which mediates a transcriptional response favouring survival, proliferation and angiogenesis. The abundance of cytokines that activate Stat3 within the tumour microenvironment, which comprises of members of the interleukin (IL) IL6, IL10 and IL17/23 families, underpins a signaling network that simultaneously promotes the growth of neoplastic epithelium, fuels inflammation and suppresses the host's anti-tumour immune response. Accordingly, aberrant and persistent Stat3 activation is a frequent observation in human cancers of epithelial origin and is often associated with poor outcome.

Here we summarize insights gained from mice harbouring mutations in components of the Stat3 signaling cascade and in particular of gp130, the shared receptor for the IL6 family of cytokines. We focus on the various feed-back and feed-forward loops in which Stat3 provides the signaling node in cells of the tumour and its microenvironment thereby functionally linking excessive inflammation to neoplastic growth. Although these observations are particularly pertinent to gastrointestinal tumours, we suggest that the tumour's addiction to persistent Stat3 activation is likely to also impact on other epithelial cell-derived cancers. These insights provide clues to the judicious interference of the gp130/Stat3 signaling cascade in therapeutically targeting cancer.

## Introduction

Chronic infection and the ensuing inflammation are among the most important epigenetic and environmental factors that contribute to tumourigenesis and the progression of established cancerous lesions [1]. Aberrant proliferation alone is insufficient to cause cancer, which requires both an initial mutagenizing event that triggers neoplastic behaviour, as well as a microenvironment that is rich in factors which support cellular survival, growth and promote angiogenesis. Many of these cytokines, angiogenic factors and chemokines are produced by activated stroma and immune cells which accumulate *in situ *during chronic inflammation [1]. As these factors not only exert profound effects on (neoplastic) epithelium, endothelial and mesenchymal cells, but also recruit immune cells, the cancer microenvironment shares many molecular features of a 'never healing wound'. In addition, tumour cells themselves acquire the ability to subvert the host's anti-tumourigenic innate and adaptive immune responses [2,3]. Accordingly, the risk of cancer development increases with the failure to appropriately resolve immune responses, which promote excessive tissue remodeling, loss of tissue architecture, and cellular stress on proteins and DNA.

Compelling evidence for a link between inflammation and cancer comes from several epidemiological studies. Chronic inflammation triggered by viral or bacterial infection increases the risk for the development of papilloma virus-associated cervical cancer [4,5], hepatitis B and C-associated hepatocellular carcinoma and Epstein Barr virus-associated lymphoproliferative disorder [6], and bacterial infections can promote metastasis following surgery [7]. In the gastrointestinal tract, Helicobacter pylori (*H.pylori*)-associated gastric cancer along with ulcerative colitis and Crohn's disease-associated colorectal cancer comprise major health issues. Besides familial adenomatous polyposis and the hereditary nonpolyposis colon cancer syndrome, ulcerative colitis accounts for one of the three highest risk groups for developing colorectal cancer [8,9]. Accordingly, the use of non-steroidal anti-inflammatory drugs (NSAIDs) and inhibitors of the rate limiting Cox-2 enzyme in the prostaglandin E2 pathway, not only inhibits chronic inflammation in patients with premalignant disease, but also reduces the risk of cancer of the colon, lung, stomach, esophagus and ovaries [10].

**Figure 1:**
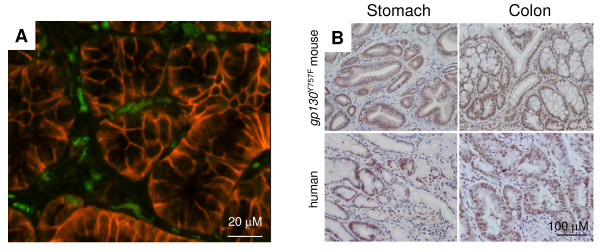
**Hematopoietic cell infiltration and STAT3 hyperactivation in gastrointestinal tumours**. **(A) **Haematopoietic cell infiltration (green) in gastric tumours of *gp130*^Y757F ^mutant mice visualized following adoptive transfer of bone marrow from GFP-transgenic mice. Metaplastic gastric epithelium is visualized following antibody binding to the intestine-specific epithelial cells surface marker gpA33 (red) [164]. **(B) **Immunohistological stain for activated STAT3 in sections of spontaneous arising gastric and CAC-induced colonic adenomas in *gp130*^Y757F ^mutant mice, and from human gastric and colonic adenocarcinomas using an antibody directed against tyrosine phosphorylated STAT3.

In recent years, studies in genetically modified mice have helped to dissect and characterize some of the underlying molecular events that link inflammation to cancer [11,12]. For instance, the development of colorectal cancer is increased in various knockout mouse models of inflammatory bowel disease [13-16], and epidemiological evidence links polymorphisms in the corresponding genes to increased inflammation and cancer susceptibility in humans. Perhaps the greatest insights, however, have been mutant mice carrying loss- and gain-of-function mutations in intracellular components where a number of oncogenic signalling cascades converge. In this review we focus on Stat3, because it provides a central signaling node for neoplastic cells to induce transcriptional responses which promote tumour growth. Stat3 is aberrantly activated in a majority of cancers of epithelial origin [17,18]. Moreover, Stat3 plays an important role in determining the outcome of the interaction between cancers and immune cells, both in terms of suppressing anti-tumour activities as well as facilitating a tumour promoting inflammatory microenvironment. These roles have recently been clarified in the gastrointestinal tract, where Stat3 has attracted attention for its capacity to functionally link inflammation to tumourigenesis (Figure 1).

## Stat3 mode of action

All seven Stat proteins act as latent transcription factors that primarily mediate signalling from cytokine and growth factor receptors. Following their activation through phosphorylation on carboxy-terminally located conserved tyrosine residues and subsequent reciprocal SH2 domain interaction, Stat proteins form stable homo- and/or heterodimers in the cytoplasm [15]. Their subsequent nuclear translocation enables binding to DNA in a sequence-specific manner and results, usually in conjunction with other cofactors, in transcriptional regulation of target genes. Different Stat proteins show preferred specificity for individual cytokine family receptors. Stat1 primarily promotes growth arrest, apoptosis, and anti-tumour immunity downstream of type I and II interferons as demonstrated by the susceptibility of Stat1-deficient mice to develop tumours [19]. By contrast, Stat3 mediates activity of cytokines generally associated with systemic acute phase and cancer-promoting inflammatory responses. Stat3 can also be activated by other cancer-associated receptor tyrosine kinases, including those for epidermal growth factor and scatter factor c-Met [20-22]. Meanwhile, cellular transformation by the cytoplasmic tyrosine kinase c-src [23] or chromosomal translocation involving the anaplastic lymphoma kinase Alk is also dependent on Stat3 [24,25]. These cytoplasmic tyrosine kinases, often in conjunction with Jaks, are likely to mediate Stat3 activation subsequent to many other cancer-initiating, toxic insults, including UV-radiation, stress, and smoke [26,27].

**Figure 2:**
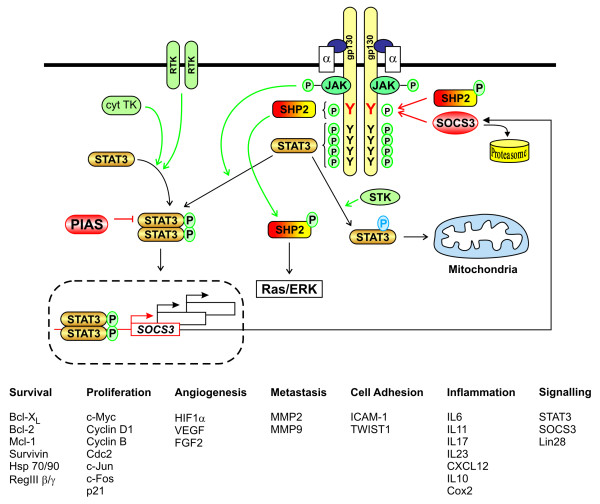
**Regulation of intracellular Stat3 signalling**. Stat3 signalling is induced by various kinases (green) in a phosphorylation dependent manner (green arrows), and counteracted by several regulatory proteins (red). For instance, Stat3 activation occurs in response to gp130 homodimerization following binding of IL6 or IL11 (blue) to their specific transmembrane receptor α-subunits (white) [165]. Phosphorylation of four membrane distal tyrosine (Y, black) residues by constitutively associated JAK-family tyrosine kinases (TK) is sufficient to enable src-homology domain (SH)-2 mediated binding of STAT3 and, to a lesser extent, of STAT1. Once tyrosine phosphorylated, STAT1/3 form homo- and heterodimers, which translocate and trans-activate target genes, including the negative regulator *SOCS3*. STAT3 can also be phosphorylated by certain cytosolic TKs and receptor TK. Meanwhile, serine threonine kinases (STK) mediate serine-phosphorylation that maximizes the transcriptional activity of STAT3 and enables its mitochondrial targeting. Gp130 also engages the Ras/ERK pathway through binding of the tyrosine phosphatase SHP2 to the membrane proximal phospho-YxxV consensus sequence (red, where V is valine and × any amino acid). This phosphor-tyrosine also provides the binding site for SOCS3 to mediate proteasomal degradation of ligand-occupied receptor complexes. Gp130 signaling is also attenuated by the Y-phosphatase activity of SHP2, while cytoplasmic PIAS3 protein sequesters Y-phosphorylated STAT3 from homodimerization, nuclear translocation and target gene activation. Representative examples of different types of *bona-fide *Stat3 target genes are listed [166].

Functionally the most important Stat3 regulators are the IL6 and IL10 family of cytokines (Figure 2). The IL6 family of ligands is defined by its shared use of the gp130 receptor β-subunit. Binding of IL6 and IL11 to their respective IL6Rα and IL11Rα receptor subunits triggers gp130 homodimerisation, while the remaining IL-6 family ligands (comprising LIF, CNTF, CT-1, Oncostatin M and IL27) induce formation of heterodimeric gp130 receptor complexes [16]. Engagement of gp130 triggers activation of the associated Janus kinases Jak1, Jak2 and Tyk2 [28,29] and subsequent tyrosine phosphorylation of gp130. While the four membrane-distal residues in the cytoplasmic tail of gp130 are required and sufficient for subsequent activation of Stat3, and to a lesser extent of Stat1, an additional membrane-proximal phospho-tyrosine residue (Y_757 _in mouse, Y_759 _in human) enables activation of the Ras/ERK pathway via the tyrosine phosphatase Shp-2. The same phospho-tyrosine in gp130 also serves as the binding site for the negative regulator Socs3, which is transcriptionally induced by Stat3. Binding of Socs3 to the activated gp130 complex results in its proteosomal degradation, thereby maintaining Stat3 activity of a transient nature. Accordingly, tissue-specific Socs3 ablation in mice amplifies ligand-dependent gp130 signalling, while the Y_757_F tyrosine-to-phenylalanine substitution in the corresponding *gp130*^Y757F ^knock-in mutant mice results in excessive activation of Stat3 and Stat1 [30,31]. Interestingly, in the context of gp130 mediated Stat activation, Stat1 and Stat3 are capable of regulating each other [32,33].

**Figure 3:**
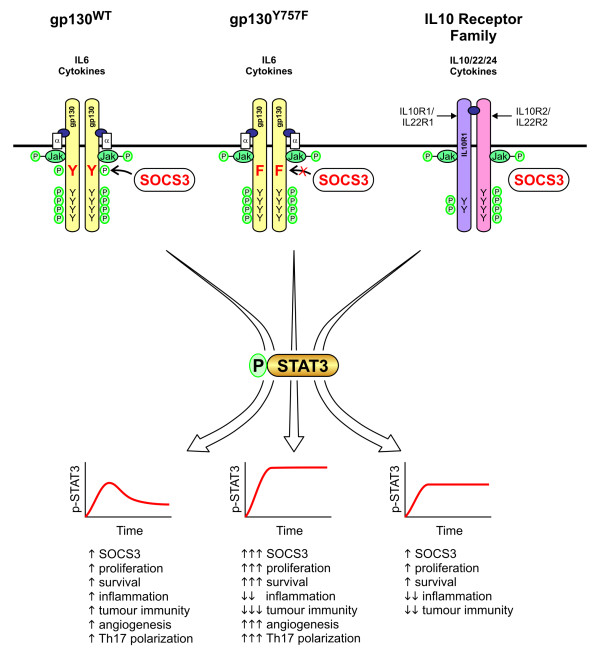
**Strength and duration of STAT3 activation determines cellular responses**. STAT3 activation patterns and associated outcomes differ between IL6 and IL10 family cytokine-induced signaling. In response to binding of ligand (blue), signaling from gp130 is transient, because the phosphorylated, membrane proximal tyrosine residue (Y, red) provides a binding site for the negative regulator SOCS3. Although *SOCS3 *is also induced in response to activation of the IL10 receptor family, STAT3 signaling remains sustained, because the IL10 receptor chains lack the corresponding YxxV motif. Similarly, signaling from the mutant *gp130*^Y757F ^receptor is sustained due to phenylalanine (F) substitution of the Y_757 _residue (Y_759 _in human GP130), resulting in exaggerated activation of its down-stream signaling molecules. The range of target genes activated differs between acute and sustained STAT3 activation, most evident in macrophages where the former promotes and the latter suppresses inflammatory response.

The IL10 family of cytokines shares the common IL10Rβ receptor subunit and comprises IL10, IL19, IL22 and IL24. IL10 confers broad anti-inflammatory responses in IL10Rα chain expressing cells, and these responses are amplified in a feed-forward loop encompassing Stat3-dependent transcriptional induction of *Il10 *[34]. Accordingly, mice lacking *il10 *or harbouring *Stat3*-deficient macrophages are characterized by excessive cytokine release and develop colitis [35,36]. IL22 is expressed during chronic inflammation by Th17, natural killer (NK) and Dendritic (DC) cells [37] and acts on IL-22R subunit expressing (intestinal) epithelial cells to induce IL10 and acute phase protein production [38]. Since the IL10-family receptor subunits lack Socs3 binding sites, IL10-mediated receptor engagement results in sustained Stat3 activation (Figure 3). Thus at least in macrophages, Socs3 provides the key molecular switch determining whether Stat3 promotes an inflammatory or anti-inflammatory response [39]. Accordingly, transient Stat3 activation by IL6 in wild-type macrophages promotes an inflammatory response, while sustained Stat3 activation by IL6 of *gp130*^Y757F ^mutant macrophages suppresses the inflammatory gene response through the induction of transcriptional repressors [39,40]. Similarly, sustained gp130 and Stat3 activation in Socs3-deficient macrophages triggers a strong anti-inflammatory response [41-43] and expression of the canonical TGFβ signaling pathway inhibitor Smad7 [31].

Since Stat3 occupies a central node for many converging signaling pathways, excessive Stat3 activity in tumours can result from oversupply of (IL6-family) cytokines and other growth factors within the tumour microenvironment. Besides these paracrine (or cell-extrinsic) pathways, activation of (proto-)oncogenes, inactivation of tumour-suppressor genes, chromosomal rearrangement/amplification and other genetic events in neoplastic cells either directly trigger Stat3 activation, or the release of inflammatory mediators as part of an autocrine (or cell-intrinsic) pathway. Remarkably, there is no genetic evidence for constitutively activating mutations within *STAT3 *itself. However, a variety of cancers harbour activating point mutations in Jak2 [44] and gp130 in-frame deletion mutations, which mediate ligand-independent activation of Stat3, are found in hepatocellular carcinomas [45]. Excessive activation of Stat3 can also result from impairment mutations affecting any of the negative regulatory proteins which limit the extent of Stat3 activation under physiological conditions [46]. For instance, epigenetic silencing of the negative regulator *SOCS3 *occurs in epithelial cancers [47], while other cancers show somatic mutations in Stat3-inactivating phosphatases T and δ [48,49]. Owing to their capacity to inactivate upstream tyrosine kinases or to sequester phosphorylated Stat3 from *de novo *Stat-dimers, mutagenic alterations in the cytosolic tyrosine phosphatases CD45 [50,51], SHP1 and SHP2 [50,51], or the SUMO E3 ligase Pias3 [52] and Grim19 [53] are also expected to result in excessive activation of Stat3-dependent target genes.

## Cellular outcomes of Stat3 activation

A decade ago, Hanahan and Weinberg have suggested that the malignant growth characteristics of cancer cells requires six essential alterations in cellular physiology, namely self-sufficiency in growth signals, insensitivity to growth inhibiting signals, evasion of apoptosis, unlimited cellular replication, sustained angiogenesis, and tissue invasion and metastasis [54]. They argued that each change represents a new capability acquired during tumour development which overcomes rate limiting steps for anti-cancer defence mechanisms in normal cells. Stat3 promotes at least three of these hallmarks (proliferation, survival and angiogenesis) and often more when investigated in specific cell types.

Stat3 inhibits apoptosis by up-regulating the pro-survival Bcl-2 proteins Bcl-X_L_, Mcl-1 and Bcl-w [55-58]. In epithelial cells, Stat3 also induces other proteins that indirectly suppress apoptosis, including the chaperone protein Hsp70 [59] and the C-type lectin-type RegIIIβ, which are both overexpressed in human colon cancer and inflammatory bowel disease [60,61] (Figure 2). In conjunction with c-jun, Stat3 inhibits the extrinsic apoptosis pathways through transcriptional repression of the FAS death receptor [62]. Stat3-mediated induction of survivin not only suppresses apoptosis, but also promotes mitogenic progression through binding to cdc2 [63,64]. However, Stat3 promotes proliferation primarily by stimulating transcription of *cyclinB1*, *cdc2*, *c-myc *and *cyclinD1 *[55,65,66], along with the induction of the immediate early genes *c-jun *and *c-fos *[67] and repression of the cell cycle inhibitor *p21 *[68]. Accordingly, Stat3 promotes the G1/S phase transition of the cell cycle in gastric, colon and squamous cell carcinoma, as well as in bladder cancer cells [65,68-70]. By contrast, *Stat3 *ablation in intestinal epithelium *in vivo *or in tumour cell lines *in vitro *resulted in cell cycle arrest in the G2/M transition and is associated with histone H3 phosphorylation-associated mitotic arrest [68].

Among the angiogenic factors, *VEGF *and *HIF1α *stand out as prominent transcriptional targets for Stat3 [71,72], and a requirement for Stat3 has been proposed for functionality of HIF1α [73]. Accordingly, Stat3 is required for endothelial cell survival and their arrangement into new vascular structures [74], while nuclear Stat3 correlates with enhanced VEGF expression and microvessel density in gastric cancer [75,76]. Since Stat3 inhibition also blocks VEGF expression in tumours characterized by aberrant activation of Src [77], therapeutic targeting of Stat3 may inhibit neovascularisation in tumours associated with excessive signaling through epidermal growth factor receptor. Stat3 may also promote neovascularisation by mediating endothelial cell responses to other growth factors, including granulocyte-macrophage stimulating factor [78]. Excessive activation of Stat3 correlates with tumour invasion and metastasis in a variety of cancers [17,18] and high level of tyrosine-phosphorylated STAT3 is a pertinent feature in colon and gastric cancers associated with adverse outcomes [79] (Figure 1). Finally, Stat3 is part of the transcriptional network that mediates epithelial-to-mesenchymal (EMT) transformation in glioblastoma [80] and promotes metastasis by induction of the extracellular matrix-degrading metalloproteinases, including MMP-2 and MMP-9 [81].

### Experimental carcinogenesis

To understand the function of Stat3 during carcinogenesis, it is helpful to divide (experimental) carcinogenesis into three distinct stages with an irreversible, genetic alteration providing the tumour initiating event. Subsequent tumour promotion occurs as a consequence of expansion of these genetically altered, pre-neoplastic cells which is frequently associated with an inflammatory response within the tumour microenvironment. Further tumour progression and growth coincides with the acquisition of additional (epi-)genetic changes, which ultimately enable the primary tumour to spread to distant metastatic sites. These sequential carcinogenesis processes can be experimentally recapitulated in mice using two-hit models employing 7,12-dimethylbenz(*a*) anthracene (DMBA) and 12-O-tetradecanoylphorbo-13-acetate (TPA) in the skin [82], or the azoxymethane (AOM) plus the polysaccharide dextran sodium sulfate (DSS) in the colitis-associated cancer (CAC) model of the colon [68]. In a hepatocellular carcinoma model, a two stage strategy is used by injecting diethylenitrosamine (DEN) as the tumour initiator and phenobarbitol as the promoter [83].

### Stat3 in epithelial cancer cells

In the CAC model, inflammation triggered through prolonged administration of DSS reveals the mutagenic effect of prior exposure to the colonotropic mutagen AOM. DSS-mediated epithelial damage and impairment of epithelial barrier function enables commensal microbes to activate resident macrophages and release inflammatory cytokines, such as IL1, TNFα and IL6. In the absence of epithelial Stat3 expression, this results in the formation of occasional low-grade intraepithelial neoplastic lesions, while epithelial Stat3 proficiency enables progression of these lesions into advanced tubular tumors [68,84]. Conversely, excessive Stat3 activation, through epithelial-specific *Socs3 *ablation or introduction of the Socs3-binding deficient *gp130*^Y757F ^mutation, results in increased tumour burden both in terms of tumour size as well as incidence [68,85]. Similar findings were obtained in the skin, where keratinocyte-specific Stat3 ablation abrogated skin tumour development [86], while keratinocyte-specific expression of the artificial, transcriptionally constitutive active Stat3C mutant, promoted the formation of squamous cell carcinoma *in situ *[82]. In either situation, Stat3 suppressed apoptosis of (mutagenized) stem and progenitor cells in the bulge region of the skin or the intestinal crypt, thereby curbing either their chance to be mutated or to subsequently expand [86] (Phesse T, Buchert M, Ernst M: Epistatic interaction between aberrant Wnt and Stat3 signaling during intestinal tumorigenesis, *submitted*). Consistent with these observations, systemic ablation of the *il6 *gene conferred a partial protective effect against tumour promotion in the CAC model, since IL6 enhances survival, proliferation and possibly cellular migration of enterocytes and their transformed counterparts that originated from the intestinal stem or transiently amplifying cell compartments [87,88]. Excessive abundance of IL6 also exacerbates colitis by suppressing apoptosis of infiltrating T-cells through "trans-signaling", whereby shedding of the extracellular domain from IL6Rα-proficient epithelium provides a soluble, ligand-binding receptor subunit for IL6 to activate gp130 in IL6Rα-deficient T-cells [89]. Thus, administration of either neutralizing IL6Rα antibodies or soluble gp130Fc suppressed enterocyte-specific Stat3 activation and proliferation, and reduced tumor incidence [90]. Concomitant overexpression of IL6 and IL6Rα in double transgenic mice is sufficient to induce hepatocellular carcinomas [91] and administration of Hyper-IL6, but not IL6, increased colonic tumours in CAC-challenged wild-type mice [84]. Due to the capacity of Hyper-IL6, a fusion protein between IL6 and IL6Rα [87,88], to activate gp130 receptors independently of the presence of the ligand-binding IL6Rα subunit, these observations suggest that cancer-initiating cells may not always express sufficient IL6Rα subunits to respond to IL6. In genetic complementation studies, we found functional redundancy between the IL6 and IL11 signaling in intestinal epithelium, where both cytokines were equally potent in conferring Stat3-dependent, epithelial resistance to DSS-induced apoptosis and colitis [68]. Consistent with these observations, IL11 administration protected against radiation-induced mucositis, suggesting that IL11 signaling may play an important role in the maintenance of intestinal epithelium [92]. Genetic deficiency for the ligand binding IL-11Rα subunit completely abrogates gastric tumour formation in *gp130*^Y757F ^mice, and mono-allelic *il11ra *ablation delayed the onset and reduced overall gastric tumour burden [32]. However, unlike the observations in the colon, gastric tumourigenesis in *gp130*^Y757F ^mice occurred independently of IL6 [32]. Meanwhile, systemic reduction of *Stat3 *expression in *gp130*^Y757F^;*Stat*3^+/- ^mice not only prevented gastric tumour formation [31], but also reduced their susceptibility to colonic tumourigenesis in the CAC model [93]. Surprisingly, *Stat1 *gene inactivation also partially reduces gastric tumourigenesis in *gp130*^Y757F ^mice [32], despite its general function in mediating IFNγ-dependent anti-tumour immunity [19]. However, therapeutic application of Stat3-antisense oligonucleotides or IL11 antagonists to *gp130*^Y757F ^mice, suggest that growth and maintenance of gastric tumours remains dependent on the continuous activation of Stat3 [31,93].

Is excessive Stat3 activation in epithelial cells sufficient to trigger *de novo *tumour formation? In models akin to (onco-)gene amplification, enforced transgenic expression of constitutive active STAT3C confers tumourigenic capacity in a 3T3 xenograph model. Overexpression of STAT3C *in vivo *also induced broncho-alveolar adenocarcinomas [94] and the formation of squamous cell carcinoma *in situ *[82] when expressed in alveolar II epithelial cells or keratinocytes, respectively. Significantly, bronchoalveolar adenocarcinomas in STAT3C transgenic mice were preceded by inflammatory cell infiltrates and tumour development was associated with excessive secretion of inflammatory cytokines, including IL6 [94]. Even though there is no evidence for tumour-specific amplification of the *STAT3 *locus in humans, excessive activation of endogenous Stat3 reproducibly promotes gastric adenoma formation in *gp130*^Y757F ^mice at a very young age. Tumour initiation and growth in this model correlates with bacterial load, because prophylactic antimicrobial treatment delayed the occurrence of these tumours [95]. Surprisingly, tumour development in *gp130*^Y757F ^mice is restricted to the glandular stomach despite systemic hyperactivation of endogenous Stat3. Consistent with this finding, we also observed that enforced, ligand-independent activation of endogenous Stat3 in the epithelium of the small and large intestine failed to confer tumour development in transgenic mice [96]. Since the *gp130*^Y757F ^germline mutation also impairs expression of the stomach-specific tumour suppressor gene *tff1 *[30], and since all colonic tumours in CAC-challenged *gp130*^Y757F ^mice harbour mutagen-induced oncogenic conversions of β-catenin, excessive activation of endogenous Stat3 may only promote tumour growth in conjunction with preexisting tumour-initiating mutation(s). However, these observations may also predicate the (co-)existence of cell type-specific Stat3 threshold levels required for neoplastic transformation, akin to those described for the canonical Wnt pathway [97,98].

**Figure 4:**
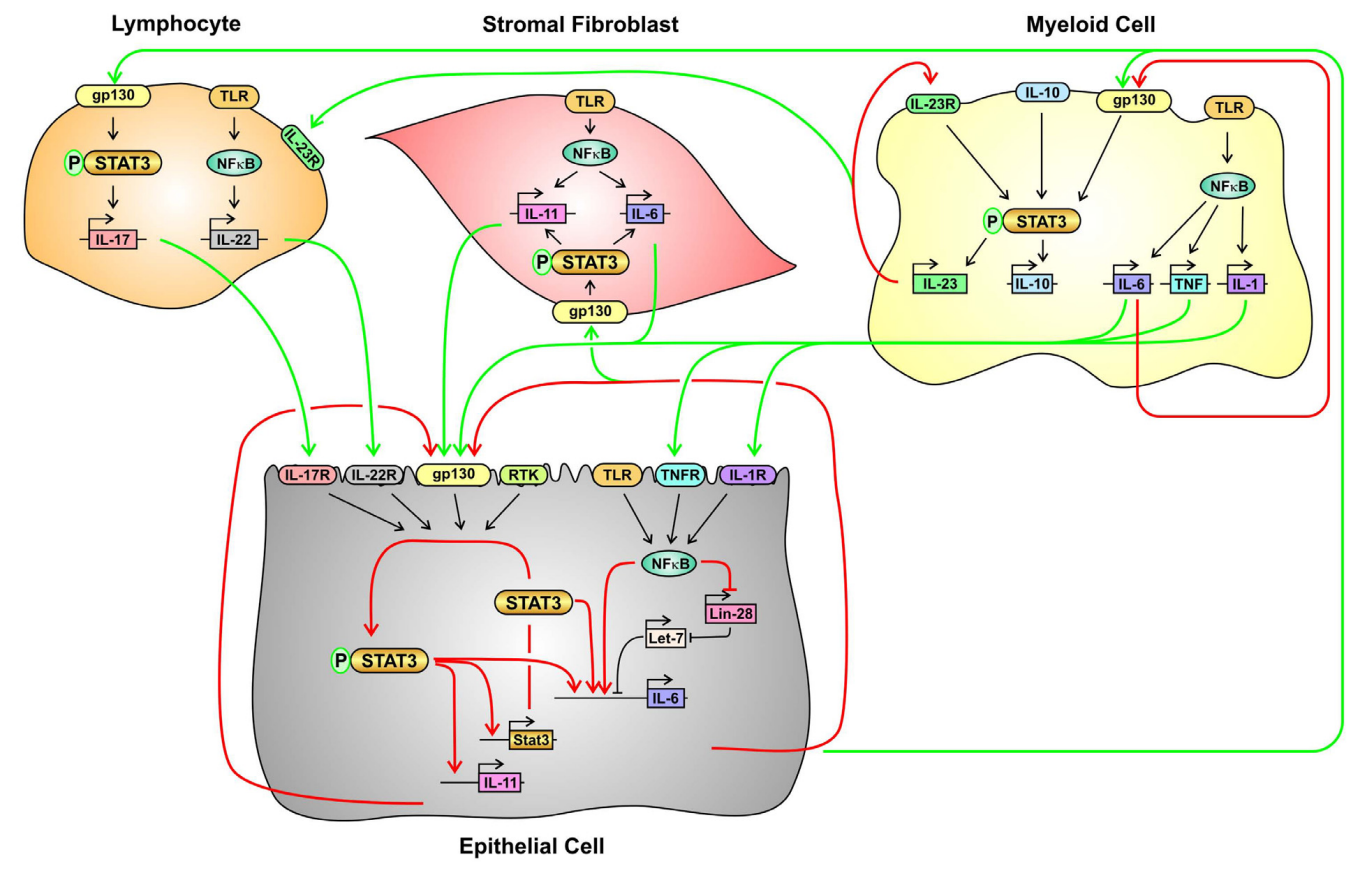
**Stat3 links inflammation to cancer**. Inflammation and cancer are functionally linked by intrinsic, Stat3-dependent autocrine feedback loops in neoplastic epithelium (red arrows) and extrinsic, feedforward and often reciprocal interactions between tumour, inflammatory and stromal cells that collectively make up the microenvironment (green arrows) [167]. The ubiquitous expression of gp130 and the capacity of STAT3 to stimulate its own transcription as well as that of gp130 ligands (in particular IL6 and IL11) also provides numerous amplification loops between the different cell types. Furthermore, limited responsiveness to IL6 and IL11 imposed by restricted expression of the ligand-specific receptor α-subunits can be overcome by IL6-transsignaling [90]. Excessive cell-intrinsic STAT3 activation is also triggered by oncogenic events from (epi-)genetic activation of positive regulators (i.e. receptor (-associated) TKs) and loss of function mutations of negative regulators (i.e. SOCS3). Expression of IL6 is also directly induced by NF-κB and indirectly through a feedback mechanism including lin28 and the let7-microRNA, while latent Stat3 in conjunction with NF-κB triggers non-canonical *IL6 *expression. Epithelial NF-κB and STAT3 are activated in response to the abundantly present inflammatory cytokines IL1, TNFα and IL6 which are released from TLR-activated myeloid cell (macrophages), with IL6 and IL11 also contributed by tumour-associated stromal fibroblast and myoepithelial cells. Meanwhile, release of IL17 and IL22 from mature Th17 cells provide an additional extrinsic link which results in excessive STAT3 activation in tumour cells.

While epithelial Stat3 activity is dispensable during normal development and tissue homeostasis of the adult intestine, reduction of Stat3 expression, by either ablating *il6 *[84] or depleting the capacity of gp130 to activate Stat3, increases susceptibility to acute colitis and impairs intestinal wound healing [30]. In humans *STAT3 *represents one of the disease loci for Crohn's and inflammatory bowel disease (IBD) [99], and most likely relates to the capacity of Stat3 to promote intestinal barrier function and integrity in response to IL6, IL11 and IL22 exposure. Expression of IL22 during chronic inflammation provides a directional signal from immune cells to epithelium, as immune cells lack IL-22R (Figure 4). Sustained activation of Stat3 in (intestinal) epithelium, brought about by activation of the Socs3-unresponsive IL10R, IL22R or *gp130*^Y757F ^receptors, induces an anti-microbial response. This comprises induction of mucins, lipocalin-2, RegIIIβ, RegIIIγ, and β-defensins to buffer the epithelium against an inappropriate innate immune response elicited by commensal bacteria and to prevent gastrointestinal inflammation and colitis [100-102]. Accordingly, experimental delivery of IL22 to mice with DSS-induced colitis reduced inflammatory infiltrates and promoted the mucosal healing response by goblet cells [103,104]. Thus, deficiency in Stat3, IL6 or IL11 signaling increases the susceptibility to colonic mucositis in CAC-challenged mice, but safeguards against excessive proliferation, survival and angiogenic activity of mutagenized cells. By contrast, the very mechanisms that confer resistance to colitis in Stat3 proficient epithelium also promote tumourigenesis, including IL22-dependent induction of tumour-promoting inducible nitric-oxide synthase [105].

Since Stat3 hyperactivation is frequently fueled by excessive IL6, an important autocrine amplification loop arises from the capacity of phosphorylated Stat3 to induce its own transcription, where *de novo *Stat3 protein in turn directly promotes expression of *il6 *without a requirement for tyrosine phosphorylation (Figure 4) [106]. The functional relevance of these autocrine and paracrine feed-forward loops, originally proposed in multiple myeloma [107] and comprising the IL6/gp130/Stat3 cascade, has recently been extended to solid tumours, including lung adenocarcinoma [20], prostate cancer [108], ovarian carcinoma [109] and Ras-transformed cancer cells [110]. In Ras-transformed cancer cells, serine phosphorylated Stat3 may also aid tumour growth by promoting metabolic functions in mitochondria possibly through its association with Grim19 [111], and stimulation of the electron transport chain in a transcription independent way [112].

While there is ample evidence for IL6 in promoting tumour activity on epithelium, the role played by the other family members is less well defined. We have identified a prominent role for non-haematopoietic IL11 rather than (myeloid-derived) IL6 in promoting gastric tumour formation in the *gp130*^Y757F ^mouse model [32]. IL11 expression correlates with development of intestinal-type gastric adenocarinoma in humans, and IL11Rα expression is linked to cancer depth and venous vessel invasion [113]. Since IL11 is expressed in epithelial and stromal cells, and its gene is transcriptionally activated by Stat3 [32], it remains to be established whether IL11 may also provide an autocrine and paracrine feed forward mechanism that, akin to IL6, fuels Stat3-dependent progression of tumours other than those of the stomach.

### Stat3 in myeloid cells

Many of the inflammatory cytokines found in the tumour microenvironment are derived from activated myeloid cells, in particular neutrophils, DC, mast cells and macrophages, where a tightly controlled Toll-like receptor (TLR)-pathway regulates the innate immune response. Excessive TLR signaling can promote tumourigenesis, since ablation of the adaptor molecule MyD88 reduced intestinal tumourigenesis in *Apc*^Min^;*MyD88*^-/- ^compound mutant mice [114]. Indeed, it has been speculated that debris from dying neoplastic cells may elicit TLR-dependent activation of macrophages or Kupfer cells in the liver [115] and engage the transcription factor NF-κB pathway through activation of its catalytic subunit IKKβ and culminating in induction of TNFα, IL-1 and IL-6 [116]. Thus, systemic administration of an IKKβ-specific inhibitor reduced Stat3 activation and IL6-target gene expression and ameliorated disease in colitis-prone IL10-deficient mice [117]. Similarly, myeloid-specific ablation of *IKKβ *inhibited tumour promotion and malignant cell proliferation in tobacco smoke- or oncogenic K-Ras-induced lung cancers [118], and reduced tumour size and multiplicity in the colon of CAC-challenged mice [11,119]. Indeed, high levels of IL6 in the tumour microenvironment are associated with the progression of colorectal [120], pancreatic [121], lung [122] and prostate cancer [123]. Furthermore, the incidence of hepatocellular carcinoma in humans, or in DEN- and CAC-induced tumours in mice, is less prominent in females due to the capacity of estrogen to suppress *IL6 *transcription [115,124]. The genetic ablation of *il6 *diminishes tumour burden in *Apc*^Min ^and in CAC-challenged wild-type mice [84,125], DEN-induced liver [115] and in a tobacco smoke-associated lung cancer model [118]. Although gastric tumourigenesis in *gp130*^Y757F ^mice occurred independently of IL6 [32], we found that *MyD88*-deficiency reduced their tumour burden (Jarnicki A, Puoczki T, Ernst M: A mouse model for innate immune cell-mediated gastric tumorigenesis, *submitted*), consistent with our observation that excessive Stat3-activation increases *Tlr4 *expression and susceptibility of these mice to lipopolysaccharide-induced septic shock (Jenkins B, Jarnicki A, Thiem S, Ernst M: Systemic alteration of IL6-mediated Stat3 signaling increases susceptibility to endotoxemia in mice, *submitted*).

Aberrant Stat3 activation in tumour cells promotes the secretion of immunomodulatory factors, which selectively reduce the Th1 dominated anti-tumour response [126]. In response to tumour-derived IL10 and VEGF, for instance, excessive Stat3 activity in myeloid cells inhibits maturation and activation within the DC lineage, favours polarization and activation of tumour associated macrophages (TAM), and reduces cytotoxic activity of neutrophils and NK cells [127]. The physical contact between tumour and antigen presenting cells also directly activates Stat3 and triggers a tolerogenic DC phenotype [128]. The capacity of Stat3 to modulate the anti-tumour immune response in macrophages and DCs partly depends on the heterodimeric IL12 cytokine family, which directs the outcome of inflammatory processes. Activation of tissue macrophages and DCs, for instance, results in production of IL12 (comprising IL12a/IL12b heterodimers) and subsequent INFγ-dependent Th1 and CTL anti-tumour responses. Meanwhile, IL10-mediated sustained Stat3 activation in TAMs represses IL12 expression and promotes production of IL23 (comprising IL23a/IL12b heterodimers), which helps to propagate the Th17 T-cell subset [129]. These findings reiterate the critical role played by Socs3 in maintaining an inflammatory, anti-tumourigenic environment characterized by IL12 expression that is converted to a tumour promoting cytokine profile when Socs3 is unable to abate gp130 signaling following engagement of the IL10 family receptor components. Accordingly, administration of Stat3 antagonists reduces tumour burden even in xenograph models where the primary tumour is not sensitive to inhibition of Stat3, suggesting that Stat3 inhibition provides a beneficial "bystander" effect on tumour cell killing that is associated with extensive tumour-specific lymphocyte infiltration [130]. Furthermore, Stat3-deficient myeloid derived suppressor cells fail to promote the formation of vessel-like structures *in vitro*, because induction of the pro-angiogenic factors VEGF, bFGF, IL-1β, MMP9, CCL2 and CXCL2 is Stat3 dependent [74]. Although, these observations suggest that excessive Stat3 activation within the myeloid cell lineages indirectly enhances tumour progression by subverting anti-tumour immunity, the contribution of myeloid Stat3 activation to the growth of tumours that are driven by persistent epithelial Stat3 activation remains less well understood. Systemic Stat3 inhibition, for instance, reduced gastric tumour burden even in *gp130*^Y757F ^mice that had undergone adoptive bone marrow transfer with wild-type cells [32].

### Stat3 in lymphoid cells

The Th17 subset of T-cells secrete large amounts of IL17A, which induces the angiogenic factors VEGF and TGFβ in fibroblasts and endothelial cells [131], and both IL17 and IL23 promote tumourigenesis [132,133]. Stat3 is indispensible for the development of the Th17 cell lineage, as it enables expression of the transcription factor RoRγt, which facilitates IL6-mediated polarization of naïve CD4 cells, and transcriptionally induces the *IL-17a *gene [134]. Thus, excessive Stat3 activity enforces differentiation into Th17 cells even in the context of Th1 polarising anti-tumour conditions [135], and genetic interference with the IL6/gp130 pathway selectively blocks Th17 cell polarization [136]. Although polarization of naïve CD4 to Th17 as well as Treg cells requires tumour-associated TGFβ in mice, only Th17 differentiation requires Stat3 activity. Accordingly, the extent of lymphocytic Stat3 activation directly shapes the overall tumour immune response including the Treg's capacity to deprive Th17 cells from essential activation cues [137]. Importantly, IL17 and IL23 alongside IL22 and cell-autonomous acting IL21, all promote and stabilize the Th17 phenotype and sustain inflammation [138] through various Stat3-dependent feed-forward loops within the tumour, stromal and haematopoietic cells of the microenvironment [133] (Figure 4). The existence of these networks are corroborated by findings that exposure of pre-neoplastic epithelium of *Apc*^Min ^mice to the enterotoxic *Bacteroides fragilis *promotes colon tumourigenesis through an IL17-/Stat3-dependent mechanism [139]. Although *H.pylori*-associated gastritis coincides with a marked mucosal induction of IL17 and IL23 [140], and these cytokines are also elevated in gastric cancer bearing *gp130*^Y757F ^mice (Putoczki T, Ernst M: A role for IL17 in a mouse model of gastric cancer, *submitted*), the latter tumours also develop in *gp130*^Y757F^;*Rag*^-/- ^mice in the absence of adaptive immune cells [141]. Indeed, the gp130-family cytokine IL-27 may promote an anti-tumour response by suppressing Th17 cell polarization and favouring Th1 differentiation through its capacity to activate Stat1 [142].

### Crosstalk of Stat3 with NF-κB and Wnt/β-catenin pathways

While Stat3 provides a major molecular link between the inflammatory response and epithelial tumourigenesis, some of its functions are also shared with NF-κB. Like Stat3, canonical activation of NF-κB induces genes that encode anti-apoptotic functions (incl. *Bcl-X*_*L*_, *Gadd45b*, *Bfl1*, *Sod2*, etc. [11,119]) to facilitate survival of (neo-plastic) cells. Therefore, inhibition of canonical NF-κB activating through ablation of the *IKKβ *gene in the intestinal epithelium decreased tumour incidence (but not size) in the colon of CAC-challenged mice [11,119]. Epithelial NF-κB activation results from the rich abundance of IL1β, TNFα and TLR-agonists in the tumour microenvironment, and IL1β, TNFα and many other cytokines and chemokines (i.e. IL6, CXCL2, CCL2 and CCL20) are transcriptional targets for NF-κB [119,143]. The intimate link between inflammation-associated hyper-activation of NF-κB and Stat3 has recently been extended by a further feed-forward loop, whereby NF-κB induction of the RNA binding protein Lin28 blocks processing of the *let-7 *microRNA (Figure 4) and thereby de-represses transcription of *il6 *[144]. It also has been suggested that Stat3 signaling prolongs nuclear retention of canonically activated NF-κB through RelA/p50 acetylation and associated interference with its nuclear export [145]. Importantly NF-κB and Stat3-mediated signaling converge on the EMT process where IL6-mediated Stat3 activation promotes EMT through transcriptional induction of the E-cadherin repressor *snail *[146], while activation of NF-κB promotes posttranslational stabilization of the Snail protein [147]. Unphosphorylated Stat3 can also cooperate with the NF-κB pathway by competing with IKKβ for binding to unphosphorylated NF-κB, and this complex activates genes, such as *rantes *and *il8*, independent of their binding sites for NF-κB and/or Stat3 [148].

While NF-κB and Stat3 cooperatively enhance survival of (neo)plastic cells through transcription of shared survival genes, the molecular mechanisms underlying functional cooperation between the aberrantly activated Stat3 and Wnt/β-catenin pathways are less clear. Evidence for the latter comes from the observation that all colonic tumours in the CAC-challenged *gp130*^Y757F ^mice harbour activating mutations in β-catenin, and that *gp130*^Y757F^;*Apc*^Min ^mice show increased tumour multiplicity [68,93], while enterocyte-specific Stat3 ablation reduced tumour incidence in *Apc*^Min ^mice [33]. Although the two pathways share transcriptional responsiveness of proliferative target genes, such as *c-myc *and *cyclinD1*, IL11 administration and excessive Stat3 activation also facilitates survival of epithelial cells with the capacity to repopulate the intestine after radiation damage [92] (Phesse T, Buchert M, Ernst M: Epistatic interaction between aberrant Wnt and Stat3 signaling during intestinal tumorigenesis, *submitted*). Similarly, Stat3 promotes survival of tissue stem cells and suppresses their differentiation [144,149] in mutagen challenged skin models and in mouse embryonic stem (ES) cells. In the fruitfly, the genes *dome*, *hop *and *Stat92E *(orthologues of mammalian gp130, Jak and Stat3, respectively) are required to reinstate gut homeostasis following apoptosis, enteric infection, or c-jun kinase (JNK)-mediated stress signaling [150]. In mammals the gene encoding intestinal Krüppel-like factor (*Iklf/Klf5*) is a target for gp130-signalling, promotes ES cell pluripotency [151] and mediates epithelial hyperplasia in the intestine [152]. Stat3 may therefore increase the pool of "stem" cells susceptible to tumour-inducing mutation, including loss-of-heterozygosity in *Apc*^Min ^mice. Moreover, the failure to eliminate cyclin D1 in situations of sustained Stat3 activation may not only bypass the DNA replication checkpoint response [153], but also facilitate aberrant chromosome segregation triggered in the absence of functional Apc protein [154].

### Targeting Stat3 activity

The preclinical observations cited above suggest that the growth and maintenance of many tumours, including some that are not caused by aberrant activation of Stat3, have become addicted to its continuous activation. However, systemic deletion of Stat3 is incompatible with embryonic development, and tissue-specific Stat3 ablation in adult mice triggers enterocolitis, impairs T-cell migration and ultimately causes Th1 autoimmunity [155]. Similarly, a dominant-negative mutation in *STAT3 *reduces its activity in human CD4 cells by approximately 75% and is associated with Hyper-IgE syndrome [156]. The latter finding is consistent with genetic observations obtained in compound mutant mice where reduction of Stat3 by more than 50% of its activity results in pathological outcomes [157]. However, systemic Stat3 haploinsufficiency suppresses growth of gastrointestinal tumours, without interfering with physiological responses during adult, fecund life [32,33]. These observations raise the exciting prospect for a therapeutic window, in which partial interference with Stat3 signaling may selectively affect tumours without the need to specifically target tumour (or tumour-associated immune) cells.

Soluble ligands have been extensively targeted by antibody-mediated therapies, and antibodies directed against IL6 and IL6Rα show promising results in the treatment of rheumatoid arthritis and other chronic inflammatory diseases. However, due to extensive redundancy among cytokines that activate Stat3, direct inhibition of Stat3 (activity) may show additional therapeutic benefits. Traditionally, pharmaceutical efforts have concentrated on targeting tyrosine kinases, and several inhibitors with specificity against Stat3-activating kinases, including EGF receptor, c-src, and Jak2, are either already in the clinic or undergoing preclinical testing [158]. These approaches are likely to be complemented by future developments of drugs that inhibit Stat3 directly. Indeed, a number of natural compounds and their derivatives, including curcumin, curcubitacins, resveratrol as well as indirubin and platinum complexes, have been shown to interfere with Stat3 activity. Their inhibitory activity most likely arises from a combination of binding directly to Stat3 as well as interfering with other cellular processes, and although compounds such as STA-21, S31-M2001 or S3I-201 suppress the growth of breast cancer, myeloma and melanoma cell lines in xenograph model, the clinical utility of these molecules still awaits confirmation. Other approaches include peptidometics and small molecules that target Stat3 dimerization, double-stranded decoy oligonucleotide to compete with Stat3 binding to target genes [159,160], as well as suppression of transcription and translation through the development of antisense-oligonuclotides [32] and small inhibitory RNA [161].

As we learn more about the underlying changes resulting from aberrant activation of Stat3, we will gain better insights into which of the aforementioned approaches may be most suitable to a particular situation. It is worthwhile to consider whether Stat3-driven tumours also develop addictions to non-oncogene pathways that are amenable to therapeutic interference [162]. Simultaneous targeting of such pathways in tumour cells, perhaps in conjunction with antibody-based strategies to curb cytokine-mediated activation of Stat3 (and NF-κB) in immune cells may hold therapeutic potential.

## Conclusions

While a link between inflammation and cancer has been known for more than a century, we now start to unravel underlying mechanisms by which chronic inflammation promotes many human cancers. Compelling recent evidence suggests that Stat3, alongside with NF-κB, acts as the signaling node which provide the functional link by which aberrant activation of inflammatory cells within the tumor microenvironment triggers an epithelial survival and growth response that promotes overgrowth of neoplastic cells. The skewed anti-inflammatory gene response elicited by prolonged Stat3 activation in myeloid cells, on the other hand, curbs the immune system's anti-tumour response, while excessive Stat3 activation in inflammatory Th17 T-cells further fuels tumour growth and angiogenesis. Persistent activation of STAT3, most prominently observed in the epithelial and immune cells that constitute the tumour invasive front, often results from autocrine and paracrine production of IL6-family cytokines by the tumour and associated stroma [143]. IL6 provides an important link between obesity, aging, chronic inflammation and cancer [163], and a wealth of genetic models now permits detailed dissection of the contribution of individual signaling components within specific cell types. A comprehensive understanding of the gp130/Stat3 signaling cascade holds great promise to identify and validate therapeutic targets that simultaneously restrict the effect of tumour promoting inflammation while restoring anti-tumour immunity.

## Competing interests

The research in the laboratory of M.E. is supported in part by a financial contribution from CSL Ltd.

## Authors' contributions

All authors have contributed to the writing of this paper.
